# Mining TCGA Database for Genes with Prognostic Value in Breast Cancer

**DOI:** 10.3390/ijms24021622

**Published:** 2023-01-13

**Authors:** Alexandru Filippi, Maria-Magdalena Mocanu

**Affiliations:** Department of Biochemistry and Biophysics, “Carol Davila” University of Medicine and Pharmacy, 020021 Bucharest, Romania

**Keywords:** breast cancer, differentially expressed genes, immune checkpoints, survival markers, immune cells

## Abstract

The aim of the study was to use transcriptomics data to identify genes associated with advanced/aggressive breast cancer and their effect on survival outcomes. We used the publicly available The Cancer Genome Atlas (TCGA) database to obtain RNA sequence data from patients with less than five years survival (Poor Prognosis, *n* = 101), patients with greater than five years survival (Good Prognosis, *n* = 200), as well as unpaired normal tissue data (normal, *n* = 105). The data analyses performed included differential expression between groups and selection of subsets of genes, gene ontology, cell enrichment analysis, and survival analyses. Gene ontology results showed significantly reduced enrichment in gene sets related to tumor immune microenvironment in Poor Prognosis and cell enrichment analysis confirmed significantly reduced numbers of macrophages M1, CD8 T cells, plasma cells and dendritic cells in samples in the Poor Prognosis samples compared with Good Prognosis. A subset of 742 genes derived from differential expression analysis as well as genes coding for immune checkpoint molecules was evaluated for their effect on overall survival. In conclusion, this study may contribute to the better understanding of breast cancer transcriptomics and provide possible targets for further research and eventual therapeutic interventions.

## 1. Introduction

Breast cancer is the number one malignancy in females all over the world, affecting approximately 13% of the female population [1] and, nowadays, as a result of advanced technology such as mammographic screening, surgery, neoadjuvant or adjuvant chemotherapy and radiotherapy approximately 70% of the disease is curable in case of early detection [2]. Nevertheless, the advanced cases with metastatic processes remain incurable with triple negative breast cancer (TNBC) having the poorest prognosis after diagnosis [3]. Because breast cancer is an international health problem, the epidemiology, tumor biology, prevention, diagnosis, prognosis, and multidisciplinary management was exceptionally well-documented [2]. Surrogate intrinsic subtypes of breast cancer, classification regularly used in clinics, includes luminal A (positive for estrogen receptors/ER+ and/or positive for progesterone receptors/PR+, negative for human epidermal growth factor receptor 2/HER2−), luminal B HER2− (ER+ and/or PR+, HER2+), non-luminal HER2-enriched (ER−, PR−, HER2+), and triple-negative (ER−, PR−, HER2−) breast cancer [2,4]. The most common subtype is Luminal A accounting for about 70% of breast cancers, followed by triple-negative (15–20%) and Luminal B (10–20%), with the HER2-enriched subtype being the rarest, at 5–15% of all cases [5]. One of the major issues regarding the neoplastic process is its heterogeneity together with the driving mutations that confer growing advantages and allow survival of the best-fit clone [6]. In breast cancer, some of the mutations are inherited breast cancer gene 1 and 2 (*BRCA1*, *BRCA2*), but most of them are acquired during the lifetime due to environment and lifestyle exposure or after chemotherapy [7,8]. Although a great progress in patient treatment has been made, after 5 years of endocrine adjuvant therapy there is a reasonable risk of breast cancer recurrence [9]. Identification of the genes responsible for long-term survival compared to short term survival will represent an asset for new therapy approaches or discovery of new biomarkers.

Cancer cells develop a series of protective mechanisms to circumvent the cytotoxic activity of the immune system and the most studied include formation of the immune checkpoints and release of the immuno-suppressive cytokines [10]. In healthy persons, these mechanisms have as main effects protection against autoimmunity and injuries caused by excessive activity of the immune system during the interactions with various pathogens [11]. The immune checkpoint complexes are formed between T cells and antigen presenting cells, which might be either immune cells or tumor cells [12]. The most studied receptors which prevent the destruction of the tumor cells by inhibiting T-cell activity are programmed cell death receptor 1 (PD1) and cytotoxic T-lymphocyte-associated antigen 4 (CTLA4) [13]. When PD1 and its ligand (PD-L1) bind together, they prevent lymphocytes to destroy cancer cells by reducing T-cell activity, proliferation, cytokine secretion and survival [14]. Immunohistochemistry studies from a large number of breast cancer cases indicated that PD1 positive samples were associated with poor prognosis [15]. Moreover, in experiments with breast cancer cell lines it has been reported that in ER-negative breast cancer cell lines (BT549 and MDA-MB-231), the mRNA and protein levels of PD1 are higher than in ER-positive breast cancer cell lines (MCF-7 and T47D) [16]. In additional experiments, survival of MDA-MB-231 breast cancer cells was increased after incubation in the presence of PD1 [17]. Several antibodies have been developed to inhibit the blockade induced by the immune checkpoints in cancer tissues [12,13].

The advance of the “omics” databases open now multiple opportunities to identify new alterations in gene expression as biomarkers and relevant targets for precision medicine. In the present work, we used transcriptomics data from The Cancer Genome Atlas (TCGA) to identify genes associated with overall survival and to characterize the tumor microenvironment in regard to the immune cells and the immune checkpoints present. The analyses are carried out with respect to the biological functions enriched in breast tumors compared to normal tissues, as well as between samples from Poor Prognosis (PP) and Good Prognosis (GP) groups of patients. We also provide a list of 742 genes differentially expressed in PP but not in GP samples and their association with overall survival.

## 2. Results

### 2.1. Demographics, Staging, and Tumor Subtypes

**Figure 1:**
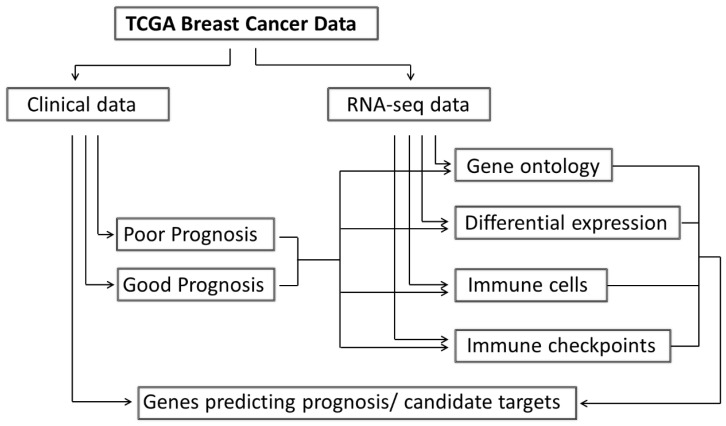
Flowchart of the bioinformatics analysis performed on publicly available transcriptomics data from TCGA.

Demographic and staging data were extracted from the clinical file downloaded from TCGA database (Figure 1).

**Figure 2:**
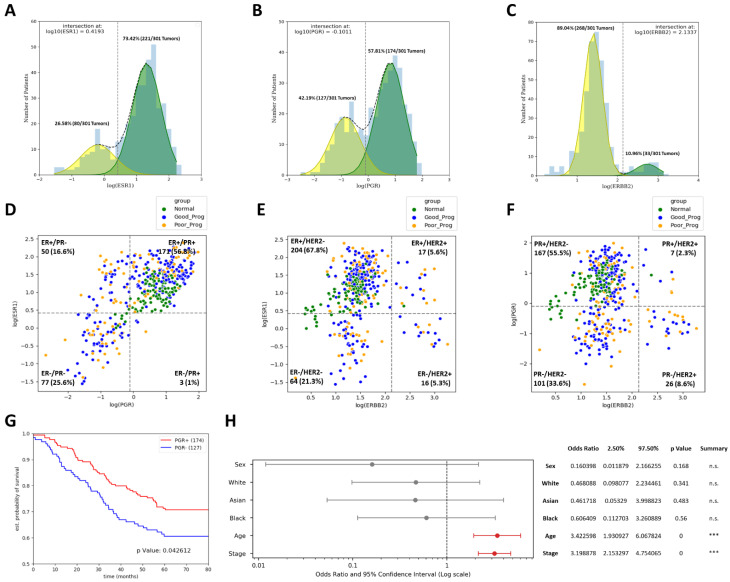
Identification of tumor subtypes based on ER, PR, and HER2 positivity. Panels (**A**–**C**) show histograms of the log10 expression profile for ER, PR, and HER2 (estrogen receptor gene/*ESR1*, progesterone receptor gene/*PGR* and, human epidermal growth factor receptor 2 gene/*ERBB2* respectively) overlaid with Gaussian curves for the positive and negative populations and the threshold values obtained. (**D**–**F**) Scatterplots showing positivity vs. negativity status for each pair of receptors. Percentages indicate proportion of all tumor samples, with normal tissues plotted for reference only. (**G**) Survival curves showing significantly better survival on tumor *PGR* positivity (*p* < 0.05, logrank test). (**H**) Multinomial logistic regression results for the clinical parameters available from TCGA. Older age and later stage were independent poor prognosis factors. Statistical significance is indicated as n.s.—not significant, *** *p* < 0.001.

Multinomial logistic regression (statsmodels package in Python3) was used to identify independent clinical prognosis factors. Table 1 summarizes clinical data according to age, tumor stage, subtype, and race according to all three group of samples investigated, namely normal tissue, good prognosis, and poor prognosis. Tumor subtypes were determined based on ER, PR, and HER2 expression profiles after establishing the positivity threshold values for these genes by Gaussian-deconvolution (Figure 2A–C). Of the 301 tumor tissues analyzed, the positivity rates were 73.42% for ER, 57.8% for PR, and 10.96% for HER2, in line with previous reports [18,19]. Based on these genes, tumor types were attributed: 68.4% of all tumors were Luminal A subtype, 6% Luminal B, 5% HER2 enriched, and 20.6% Triple Negative. Patients in the PP group had slightly higher Triple Negative (24/101 (23.8%) vs. 38/200 (19%)) and luminal B percent (9/101 (8.9%) vs. 9/200 (4.5%)) compared with GP tumors; however no significant variation was observed (see Table 1).

Survival effect of the three receptors was assayed and patients with ER+ and HER2− had slightly better survival (Supplementary Figure S1); however, only PR+ status yielded significantly better survival (survival fraction at five years: 0.75 vs. 0.61, *p* = 0.0426) compared with PR− (Figure 2G). Breast cancer samples investigated for positivity or negativity of ER, HER2 and PR (Figure 2D–F) with the highest expression in the following receptor combinations ER+/HER2− (67.8%), PR+/HER2− (55.5%) and ER+/PR+ (56.8%). Interestingly, when PR vs. ER expression status was assayed, only three tumors fell into the ER−/PR+ category and quite close to the borders of this region, suggesting that ER−/PR+ category might indeed be a measuring artifact and not a distinct pathological entity, as others have already proposed [20]. The lowest percentages identified in case of two receptors analysis are in case of ER−/HER2+ (5.3%), PR+/HER2+ (2.3%), and ER−/PR+ (1%).

Age over median (56 years) and advanced stage proved to be independent prognosis factors increasing likelihood of death by a factor of three each, while sex and race did not significantly affect prognosis (Figure 2H).

### 2.2. The Most Significant Differences between GP and PP Samples Are Interferon Gamma Signaling and Anti-Tumor Immune Response

**Figure 3:**
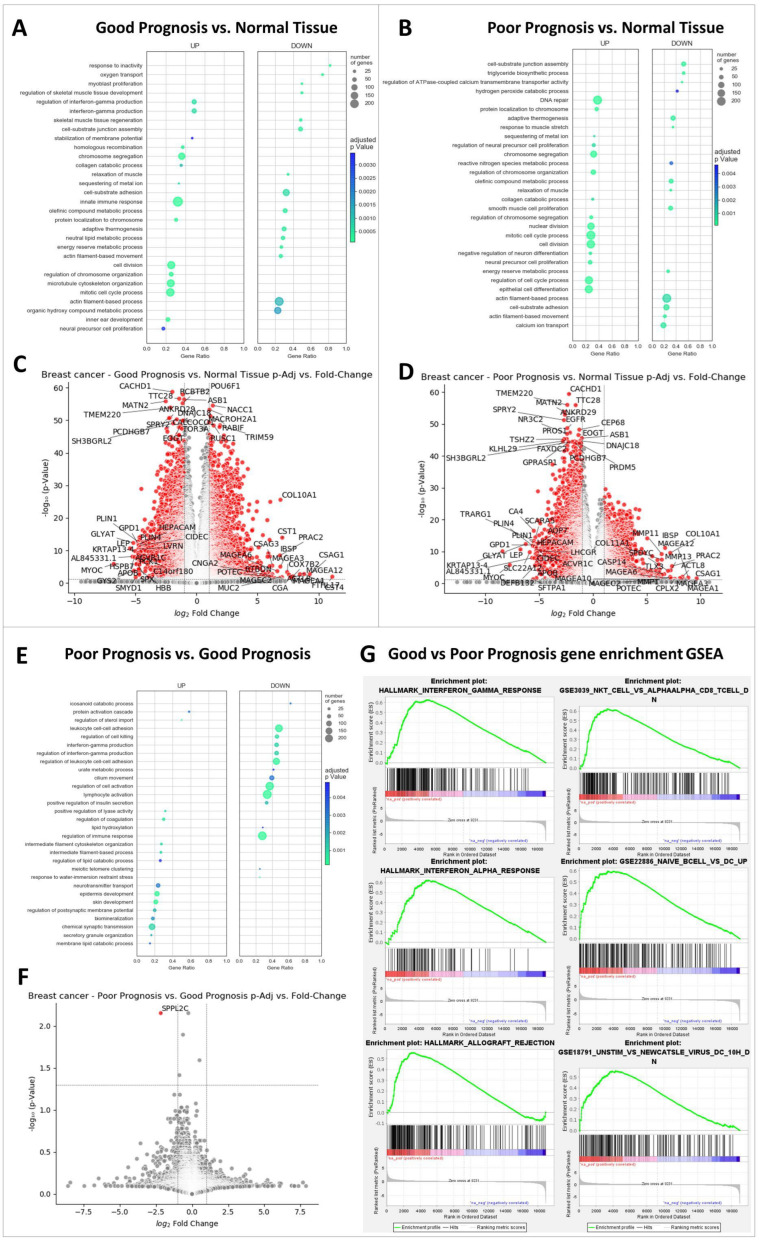
Differential expression and gene ontology. (**A**,**B**) Plots showing enriched gene sets in tumor samples from the GP and PP group, respectively, as compared with normal tissue. Circle sizes reflect the number of genes enriched, the *x* axis the proportion of all genes in that specific gene set that are enriched and the color denotes the FDR adjusted p value. (**C**,**D**) Volcano plots of differential expressed genes between GP tumors vs. normal tissue and PP tumors vs. normal tissue, respectively, showing log(FoldChange) plotted on the statistical significance (FDR adjusted p value). Most significantly modified genes, most under-expressed, and most over-expressed 20 genes are labeled. (**E**) Diagram that shows the results of PP vs. GP gene enrichment analysis. (**F**) Volcano plots of differential expressed genes in PP tumors vs. GP tumors. After Benjamini Hochberg p value correction for FDR, only one gene remains significantly modified between the two groups. (**G**) Most enriched gene sets in the GP group compared with the PP group from the “Hallmark” and “C7: immunologic signature genes” collections from GSEA.

Gene ontology results revealed that the biological processes primarily enriched in the tumors from patients with poor survival were mainly related to cell cycle regulation, cell division, and DNA repair, while biological processes related to the normal metabolic function such as triglycerides synthesis, thermogenesis, calcium transport, reactive species removal were depleted along with cell-substrate interactions. Similarly, tumors from patients with better survival were also enriched in genes related to cell cycle regulation and cell division, but crucially, also in processes related to anti-tumor immunity: interferon gamma production and innate immunity (see Figure 3A,B). This difference between tumors from patients in the PP group vs. GP group became even more apparent when the groups were directly compared and was found that PP tumors were significantly less enriched in genes involved not only in interferon gamma production and regulation of immune response, but also in leukocyte cell–cell adhesion, lymphocyte activation, and cell killing (Figure 3E).

A different gene set enrichment analysis performed on the 50 hallmark gene sets revealed significantly enriched (after the false discovery rate (FDR) 1% correction of the p values) interferon gamma response, interferon alpha response and allograft rejection gene sets in the GP vs. PP group. Gene set enrichment analysis (GSEA) was also performed on the 5086 immunologic signature gene sets and the most GSEA gene sets enriched were related to responses in NK cells, B cells, and dendritic cells (Figure 3G).

### 2.3. Highly Expression of MAGEA Family Members, PRAC2, CSAG1, and COL10A Gene Profiles in Breast Cancer Samples

To compare differentially expressed genes in normal tissue with GP and PP breast cancer, 20 most significantly modified genes, most under-expressed and most over-expressed genes have been plotted as significance versus fold changed using volcano diagrams (Figure 3C,D,F). Gene expression profiles were evaluated for both PP and GP samples against normal tissues. The results showed highly expressed genes encoding for melanoma-associated antigens A (*MAGEA*) family members, prostate, rectum and colon expressed protein 2 (*PRAC2*), chondrosarcoma-associated protein 1 (*CSAG1*), and collagen type X alpha 1 chain (*COL10A1*) in both PP and GP samples. Nevertheless, several differences are identified in GP compared to PP, namely mucin 2 (*MUC2*) and chorionic gonadotropin alpha chain (*CGA*) genes are over-expressed in GP, while matrix metalloproteinase (*MMP*) family members and actin-like 8 (*ACTL8*) genes are in PP.

### 2.4. Gene Signature and Survival in Patients with Breast Cancer

**Figure 4:**
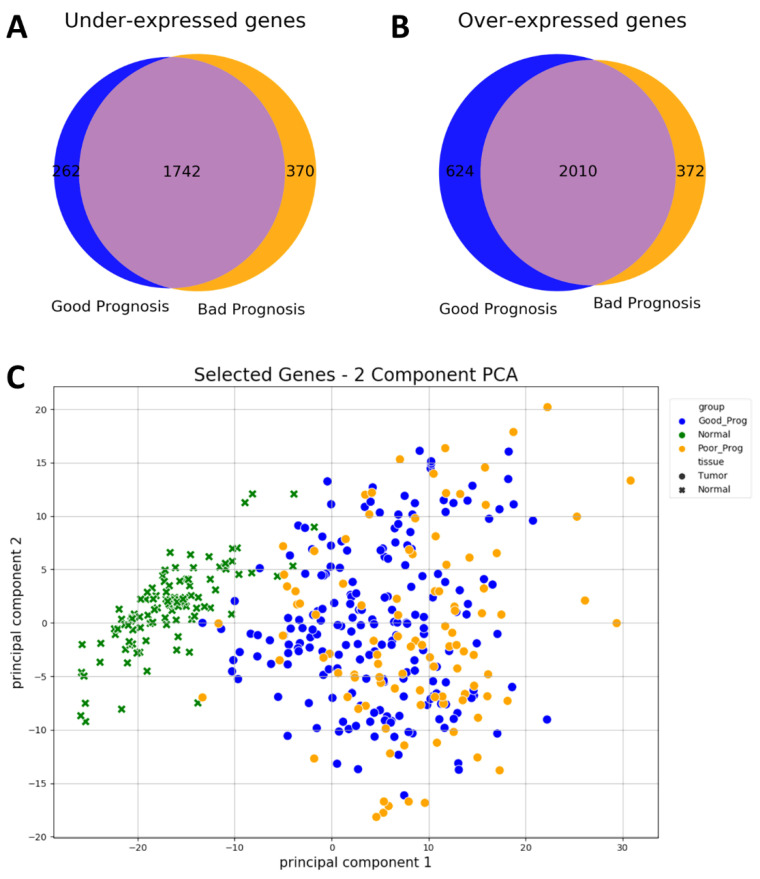
Comparison of under- and over-expressed genes in GP and PP breast cancer samples. Overlap in under-expressed genes (**A**) and over-expressed genes (**B**) between PP and GP samples, as compared to normal tissues. 370 genes were significantly under-expressed in PP tumors but not in GP tumors, while 372 genes were over expressed in PP tumors but not in GP tumors. These genes were used for the 2 component—principal component analysis illustrated in (**C**). An almost perfect separation was observed between normal and tumor tissues, but not between GP and PP samples.

As differential expression of genes between PP and GP groups yielded only one significantly modified gene after FDR correction of p values by Benjamini Hochberg method, we compared the overlap of under- and over-expressed genes in each group compared to normal tissue. In GP, 2004 genes were found to be significantly under-expressed, while 2634 were over-expressed compared to normal tissues, and PP showed similar numbers: 2112 genes under-expressed and 2382 over-expressed. We selected for further analysis those genes that were under-expressed in PP but not in GP (*n* = 370) and over-expressed in PP but not in GP (*n* = 372). These genes were able to separate normal tissues from tumor tissues in a principal component analysis, however, the PP and GP populations overlapped, see Figure 4.

**Figure 5:**
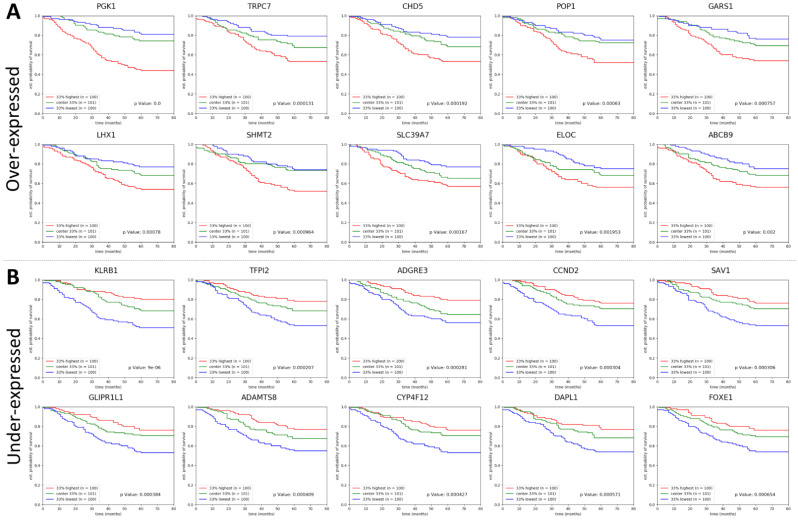
Association between over- and under-expressed genes in PP breast cancer and overall survival. Survival curves showing the 10 most significantly associated with survival genes from the 372 overexpressed genes (**A**) and from the 370 under-expressed genes (**B**). Expression values for each gene were sorted and patients were divided in 33% highest levels of expression, center 33% and 33% lowest expression levels. *p* Values indicated in the graphs represent the statistical significance of the top 33% compared with the bottom 33% survival by logrank tests and the results were visualized using the lifelines, sksurv, and matplotlib Python packages.

While the most significant effect on survival for the canonical ER and PR status was obtained for ER+/PR+ (survival fraction at five years: 0.7) versus ER+/PR− (survival fraction at five years: 0.56), with a *p* value < 0.05, some of the selected genes seem to offer a much better differentiation between poor and good prognosis. The most significant association was obtained for the phosphoglycerate kinase 1 gene (*PGK1*) overexpressed in PP but not in GP, yielding a survival fraction of about 0.81 for the 1/3 of patients with low expression levels vs. about 0.44 for the 1/3 of patients with high expression levels (*p* < 0.0001). Similarly, among the under-expressed genes in PP samples, the most associated with survival was killer lectin-like receptor B1 gene (*KLRB1*) with a survival fraction at 5 years of 0.81 for the 1/3 of patients with high expression levels vs. 0.51 for the 1/3 of patients with low expression levels (*p* < 0.0001). Figure 5 shows the ten most associated with survival over-expressed and under-expressed genes and the full list of the selected genes and their impact on survival can be found in Supplementary Table S1.

### 2.5. High Levels of Pro-Inflammatory Macrophages and Cytotoxic T Cells, While Lower Levels of Anti-Inflammatory Macrophages Are Found in Tumor Samples Compared to Normal Tissue

**Figure 6:**
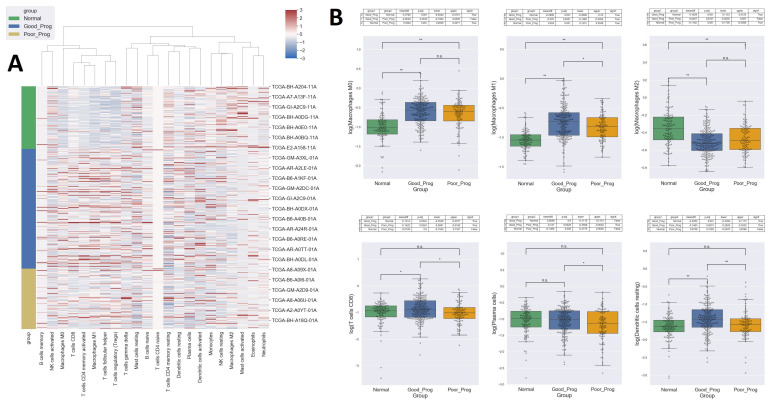
Cell populations enrichment. Heat map showing the results of CIBERSORTx after z Score normalization with columns grouped by hierarchical clustering based on the ward distance and rows showing sample groups (**A**). Box plots of the relative cell enrichment by sample group showing selected cell types. Statistical significance is indicated as n.s.—not significant, * *p* < 0.05, ** *p* < 0.01 (**B**).

Cell enrichment analysis performed in CIBERSORTx with the signature matrix containing 22 functionally human immune subsets (LM22) revealed a different makeup of immune cells in normal and tumor tissues with significantly more non-polarized M0 macrophages, pro-inflammatory M1 macrophages, cytotoxic T cells, and less anti-inflammatory M2 macrophages in tumor tissues compared with normal tissues. T-cells follicular helper and regulatory T cells were also increased in both PP and GP samples compared to normal tissues but no significant differences were observed between the two subsets of tumors. Moreover, PP samples had fewer cytotoxic T cells (*p* < 0.05), plasma cells (*p* < 0.05), dendritic cells (*p* < 0.01), and M1 macrophages (*p* < 0.05) and tended to have more M2 macrophages (although not statistically significant), compared to the GP samples (see Figure 6).

### 2.6. Identification of Immune Checkpoint Molecules Associated with Breast Cancer

**Figure 7:**
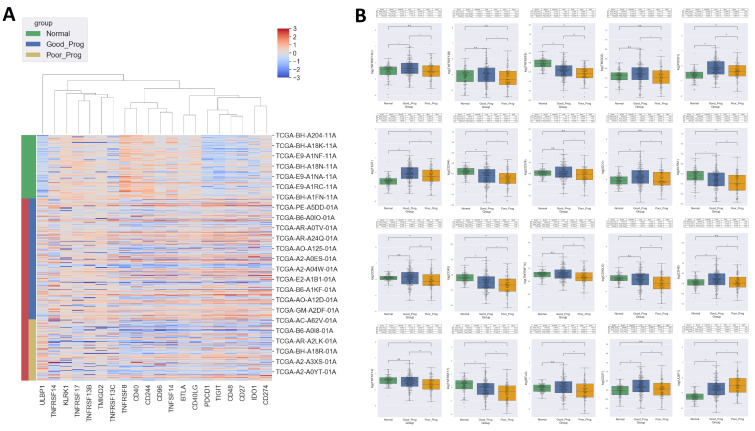
Heat map showing the immune checkpoints significantly overexpressed or underexpressed in PP vs. GP. Data were z score normalized, columns were grouped by hierarchical clustering based on the ward distance and rows showing sample groups (**A**). Box plots of the relative gene levels by sample group. Statistical significance is indicated as n.s.—not significant, * *p* < 0.05, ** *p* < 0.01 (**B**).

A list of 75 immune checkpoint molecules was obtained from Charoentog et al. [21] and 67 were identified in the analyzed samples. Of those, 20 molecules had differential expression between PP and GP samples (Figure 7):

(i) 6 genes encoding for immune-inhibitors had lower values in PP: programmed cell death protein 1 (*PDCD1*), B and T lymphocytes associated protein (*BTLA*), T cell immunoreceptor with Ig and ITIM domains (*TIGIT*), indoleamine 2,3-dioxygenase 1 (*IDO1*), clusters of differentiation 96, 244 (*CD96*, *CD244*),
(ii) 13 genes encoding for immune-stimulators had lower values in PP: clusters of differentiation 27, 48, 40, 40LG, 274 (*CD27*, *CD48*, *CD40*, *CD40LG*, *CD274*), killer cell lectin like receptor K1 (*KLRK1*), transmembrane and immunoglobulin domanin containing protein 2 (*TMIGD2*), TNF receptor superfamily members 8, 13B, 13C, 14, 17 (*TNFRSF8*, *TNFRSF13B*, *TNFRSF13C*, *TNFRSF14*, *TNFRSF17*), TNF superfamily member 14 (*TNFSF14*), and
(iii) 1 gene encoding for an immune-stimulator had greater expression in PP: UL16 Binding Protein 1 (*ULBP1*).

**Figure 8:**
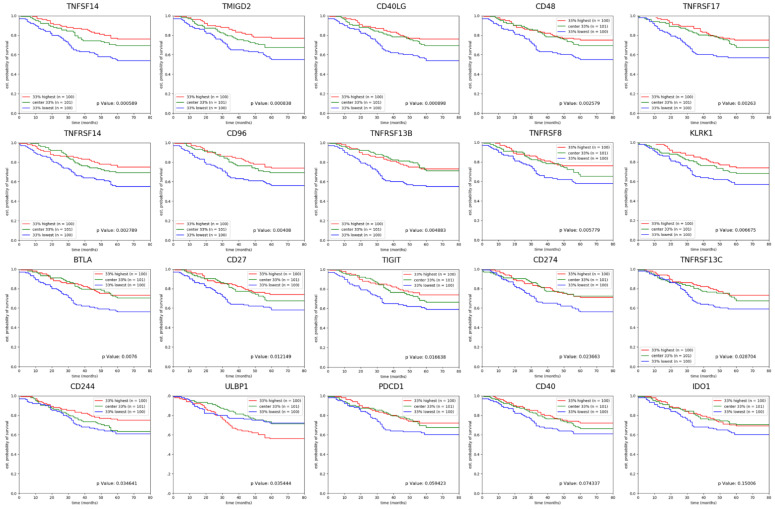
Survival curves showing the survival impact of the 20 immune checkpoint genes with altered expression in PP compared with GP. Expression values for each gene were sorted and patients were divided in 33% highest levels of expression, center 33% and 33% lowest expression levels. *p* Values indicated in the graphs represent the statistical significance of the top 33% compared with the bottom 33% survival by logrank tests and the results were visualized using the lifelines, sksurv and matplotlib Python packages.

The survival curves for different expression levels of these immune checkpoint molecules were plotted and can be found in Figure 8.

## 3. Discussion

In this study, we investigated heterogeneity, gene expression profiles, and activation of immune system (activated macrophages, T cells, and immune checkpoints) in breast cancer samples using transcriptomics data. We analyzed 406 samples, divided in 105 control and 301 breast cancer samples, out of them 200 samples with good prognosis (greater survival than five years) and 101 with poor prognosis (survival less than five years). Breast cancer samples were further attributed molecular subtypes based on their receptor expression profile. An advantage of using continuous expression data, such as that obtained from RNA-seq, in addition to the classic immunohistology method is that no subjective grades are introduced and the thresholds for positive vs. negative expression are more precise. 

We report here a series of modified genes identified in breast cancer samples from patients with different survival outcomes. The GP samples are associated with over-expressed genes, such as gene encoding for human mucins, PP samples with genes encoding for matrix metallo-proteinases, while both groups included genes encoding for cancer/testis antigens (e.g., *MAGEA*) or short collagen chains (e.g., *COL10A*). Out of the 742 genes analyzed for overall survival, over-expression of *PGK1* gene and down-regulation of *KLRB1* gene were the best candidate markers for breast cancer with poor prognosis. Reduced interferon gamma signaling was associated with decreased immune responses in PP samples, with fewer cytotoxic T cells, plasma cells, dendritic cells, and M1 macrophages, compared to GP samples. We also provided 20 differentially expressed immune checkpoint molecules between PP and GP samples as well as other putative target molecules to be used in the therapy or diagnosis of breast cancer.

While some authors point to ER−/PR+ tumors as being a distinct pathological entity, with responsiveness to therapies and clinical outcomes [22], there are others showing that retesting tumors initially diagnosed by immunohistochemistry (IHC) as ER−/PR+ might change the diagnostic when more precise methods are used [20,23]. This controversy also stems from the fact that PR expression is under the regulation of ER as a transcription factor [24] and thus, lack of ER should limit, if not preclude, PR expression. We show here that when plotting ER expression vs. PR expression (see Figure 2D), the scatter plot follows a diagonal distribution, with only three tumors present in the ER−/PR+ quadrant, with both ER and PR levels close to the determined positivity thresholds. This observation is in contrast to the plots with samples for ER vs. HER2 (Figure 2E) or PR vs. HER2 (Figure 2F) where all respective four possible combinations show distinct populations. As those three ER−/PR+ tumors represent less than 1% of all 301 tumors studied, and the expression levels of both ER and PR are close to the thresholds, our results question the clinical significance of the ER−/PR+ classification. Demographics, staging, and subtypes data demonstrated that late stage and advanced age were associated with breast cancer poor prognosis results, in line with the previous reports [22].

Gene ontology (GO) analysis identified several sets of genes involved in the activity of immune system and cell cycle (including microtubule activity) that are overexpressed in GP samples compared to the normal tissue, whereas other sets of genes responsible for production of proteins involved in cell substrate adhesion and actin are downregulated. 

**Figure 9:**
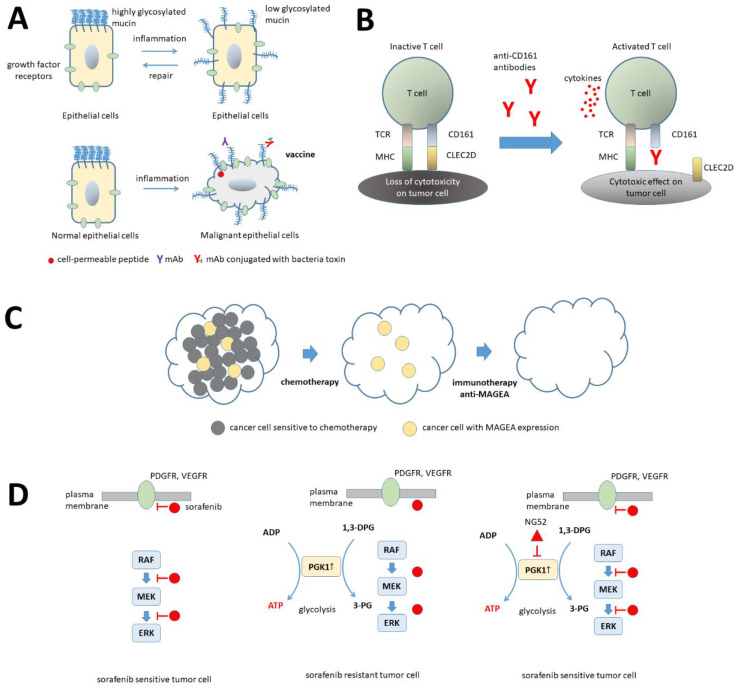
Therapeutic targets in breast cancer. (**A**) MUC1 molecule. Epithelial cells display polarity with highly glycosylated mucin in the apical pole and growth factor receptors in the baso-lateral areas. Stress factors, such as inflammation, induce loss of polarity associated with redistribution of mucin from the apical pole to baso-lateral areas and redistribution of growth factor receptors from baso-lateral areas to apical level. Nevertheless, in normal epithelial cells the process is reversible. In cancer cells tight junctions are destroyed, the low glycosylate mucin molecules interact with growth factors and cell polarity is lost, leading to increased epithelial permeability. Several attempts have been made to target mucin molecules overexpressed on the surface of cancer cells and to reduce their interactions with growth factors receptors: cell-permeable peptides, monoclonal antibodies (mAb) against mucin molecules, mAb conjugated with bacteria toxins or vaccines [25,26,27,28]. (**B**) CD161 receptor. Tumor cells express CLEC2D ligand for CD161 receptor and inhibit cytotoxic T-cell activity. Inhibition of CD161 receptor by antibodies leads to antigen recognition by T cells and increase their cytotoxic activity by cytokine release [28]. (**C**) MAGEA antigens. Malignant tumors may contain chemo-sensitive cells and apoptosis resistant MAGEA cells. Chemotherapy administration can destroy most of the cancer cells, but not MAGEA positive cells. After chemotherapy, immune-targeted therapy against MAGEA can be addressed [35,36]. (**D**) PGK1 enzyme. Malignant cells are sensitive to a small inhibitor kinase, sorafenib that can act on PDGFR, VEGFR, or RAF/MEK/ERK pathway. Nevertheless, cancer cells acquire drug resistance to sorafenib associated with high expression of PGK1. Addition of NG52, cell cycle-regulated kinase inhibitor might inhibit PGK1 activity and restore sensitivity of cancer cells to sorafenib [37,38,39]. Legend: MUC1, mucin 1; MAGEA, melanoma associated antigen A; KLRB1, killer cell lectin-like receptor B1 or CD161; PGK1, phosphoglycerate kinase 1; 1,3-DGP, 1,3-diphosphoglycerate; 3-PG, 3-phosphoglycerate.

One of the overexpressed genes in GP and not in PP breast cancer samples was *MUC2*, responsible for production of mucin single pass transmembrane glycoprotein 2. The most studied mucin isoform is human mucin 1 (MUC1), which in normal epithelia from breast, esophagus, stomach, duodenum, pancreas, lung, uterus and prostate is localized in the apical area having the role to protect normal tissue against toxins, microorganisms, viruses, or low pH [25]. In epithelial cancer cells, mucins, aberrantly glycosylated and overexpressed, are responsible for induction of proliferation (in collaboration with growth factors), aberrant glucose metabolism, destabilization of cell junctions and drug resistance [26]. Since mucins are involved in the progression of malignancy, they might be considered cancer biomarkers for staging or relapse after therapy and target for therapy. Figure 9A showed redistribution of mucin molecules and the ability of normal epithelial cells to recover after the interaction with inflammatory stimuli, whereas malignant cells do not recover. Several therapeutic attempts are in use to reduce the negative influence of mucin molecules in malignancy [25,26,27,28].

*MAGEA1/3/12/13*, *MMP11/13*, *PRAC2*, *CSAG1*, *COL10/11A1* genes are overexpressed in breast cancer samples from patients with PP (Figure 3). *MAGEA* genes are responsible for production of melanoma-associated antigens A (MAGEA), a class of cancer/testis antigens expressed in both normal germ lines and malignant cells [29]. MAGEA are present at high levels in several tumor tissues, such as colon, melanoma, brain, prostate, breast [30] and their pathological activity is associated with increased motility, inflammation, and resistance to apoptosis [29]. Moreover, MAGEA2 was identified as molecule involved in tamoxifen-resistant breast cancer [31]. All this characteristics make from MAGEA a good candidate as biomarker and target for immunotherapy (Figure 9). *MMP* genes, which encode for matrix metalloproteinases (MMP), in normal tissues are involved in several physiological processes, such as development, growth or immune responses [32], whereas in cancer tissue MMP activity is correlated to extracellular matrix substrates degradation, neovascularization, and metastasis [33]. Small inhibitors and antibodies have been designed to interfere with MMP activity in malignant transformation [32]. Our data regarding overexpression of MMP in breast cancer samples corroborate with previous published reports [34].

Collagen type X alpha 1 chain (COL10A1) encoded by *COL10A1* gene is a member of the collagen family, whose activity was associated with promotion of metastasis through epithelial-mesenchymal transition and poor survival in gastric [40] and colorectal cancer [41]. Again, our data that show up-regulation of *COL10A1* gene in PP breast cancer samples are in line with previous published results in other pathologies. *PRAC* gene is normally expressed in the gastrointestinal tract, prostate, testis, urinary bladder, vagina, and placenta but not in breast tissue and, similarly, *CSAG1* expression should be limited to brain and testis tissues [42], thus, their expression in breast tissues, is highly indicative of cancer.

Survival analysis has been carried out for a selection of 742 genes upregulated or downregulated in PP breast cancer. The upregulated genes most associated with survival were: *PGK1*, short transient receptor protein potential channel 7 (*TRPC7*), chromodomain helicase DNA binding protein 5 (*CHD5*), processing of precursors 1 (*POP1*), glycyl-TRNA synthetase 1 (*GARS1*), LIM homeobox 1 (*LHX1*), serine hydroxymethyltransferase 2 (*SHMT2*), solute carrier family 39 member 7 (*SLC39A7*), elongin C (*ELOC*), ATP binding cassette subfamily B member 9 (*ABCB9*). The downregulated genes most associated with survival were: *KLRB1*, tissue factor pathway inhibitor 2 (*TFPI2*), adhesion G protein-coupled receptor E3 (*AGRE3*), cyclin D2 (*CCND2*), Salvador family WW domain containing protein 1 (*SAV1*), GLI pathogenesis related 1 like protein 1 (*GLIPR1L1*), a disintegrin and metalloproteinase with thrombospondin motifs 8 (*ADAMTS8*), cytochrome P450 family 4 subfamily F member 12 (*CYP4F12*), death associated protein like 1 (*DAPL1*), and forkhead box E1 (*FOXE1*). *PGK1* gene codifies for phosphoglycerate kinase 1 (PGK1), which is considered survival biomarker and invasion promoter in breast cancer [43,44]. PGK1 is involved in glucose metabolism, being responsible for the conversion of 1, 3-diphosphoglycerate to 3-phosphoglycerate. In transformed cells, it is highly expressed in ovarian cancer [45]. Beside the above-mentioned activities, PGK1 is responsible for drug resistance in malignancy, however the exact mechanism is not known [37]. A possible mechanism to overcome drug resistance induced by sorafenib in cancer cells with high PGK1 expression might be the inhibition of the kinase with small inhibitors (Figure 9). Another gene with significance in overall survival for patients with PP breast cancer is *KLRB1*. This gene is responsible for production of killer cell lectin-like receptor B1 (KLRB1) or CD161 receptor, which is expressed on natural killer, CD8+ and CD4+ cells [46]. CD161 receptor was found to be downregulated in 13 types of cancers [47], our data regarding PP breast cancer samples being in line with previous reports. In some types of cancer (i.e., TNBC, androgen-independent prostate cancer, glioma), CD161 appears to have immunosuppressive role, since tumor cells express C-Type Lectin Superfamily 2, Member D (CLEC2D) ligand and interaction between CD161 and CLEC2D hinders the cytotoxic activity of T cells [48,49]. Inhibition of this communication by anti-CD161 antibodies allowed T cells, through TCR-MHC complexes and cytokine release, to destroy the tumor cells (Figure 9). 

The variances in prognosis associated with different molecular subtypes of breast cancer may arise partly from different responsiveness to treatment (Supplementary Figure S1), with hormone positive cancers (Luminal A > Luminal B) being the most responsive [50,51]. The cases archived in the TCGA database span a period from 1988 to 2013, with a fair number of cases dating from before FDA approval of tamoxifen as a first line of treatment in 1998 [52]. In the data presented here, only one patient has recorded treatment with hormone therapy, immunotherapy, or targeted molecular therapy. Thus, the lack of prognostic value observed in this study for the different molecular subtypes is probably a consequence of the absence of targeted treatments (Supplementary Figure S1A). Moreover, HER2 positivity led in a previous study to a 5-year survival rate of about 65–70% [53], similar to the 61% rate observed here (Supplementary Figure S1B). A lack of predictive value for the HER2 enriched subtype in presented results could therefore stem from the relatively low number of identified cases with this subtype (*n* = 15; 5% of all tumors). Triple negative breast cancers (TNBC) are known to lead to a poor prognosis, with reported overall 5-year survival rates between 62% and 82%, compared to 75% to 86% in the case of non-TNBC patients [54,55]. Our results agree with the survival rates of TNBC patients, the data analyzed here yielding a 5-year survival rate of 61% (Supplementary Figure S1C). However, the results presented here show lower than 5-year overall survival of patients with non-TNBC tumors than the previously published data (only 68% compared to the previously published 75% to 86% interval).

Because the PP group contained a significantly larger fraction of advanced tumors compared to the GP group (Table 1, stage III and IV), it is possible that some of the differences in the expression profiles may reflect the immune system failing to mount a proper immune response in patients whose general health condition declines due to the tumor spreading. When a PCA analysis was performed using the 742 differentially expressed genes analyzed in this study, tumors from different stages overlapped (Supplementary Figure S2), suggesting that the identified genes are not stage-specific. The fact that different molecular subtypes of breast cancer could have substantially different gene expression signatures could decrease the statistical power of identifying genes associated with prognosis when studying all subtypes together as it was done in this study. Thus, the analysis of the genes found to be associated with prognosis might not be complete; however, it could serve as a starting point for further studies. Moreover, in contrast with individual genes, it is plausible that the tumor immune escape described here based on gene sets is a common negative outcome in all molecular subtypes. It should also be noted that, because the data analyzed contained a larger proportion of Luminal A tumors compared to other subtypes, the results could be biased toward finding prognosis markers in this subset of tumors.

GO analysis can identify gene sets enriched in any of two groups, comparing one against the other but is almost always performed between tumor and normal tissue samples in order to find biological functions altered in tumors compared to healthy tissues [56,57,58]. In order to better characterize the tumor microenvironment, we extended that use to also identify the biological functions significantly altered in PP compared to GP samples. GO analysis revealed Interferon Gamma Production and Regulation of Interferon Gamma Production as some of the most significantly altered biological functions in PP vs. GP samples, as well as reduced related biological functions such as Lymphocyte Activation and Regulation of Immune Response in PP samples. A subset of dendritic cells (DC), plasmacytoid dendritic cells, is one of the main producers of type I interferons (IFN) acting to support CD8+ cytotoxic T lymphocytes, inactivate the suppressive function of regulatory T cells [59] and also to polarize macrophages toward the M1 pro-inflammatory phenotype [60]. Our results not only show tumors from GP patients to be significantly enriched in genes associated with IFNγ production compared to their PP counterparts, but also higher DC, M1 macrophages, and CD8+ T cells numbers elicited by the IFNγ actions, as revealed by cell enrichment analysis performed with CYBERSORTx. Moreover, macrophage M1 and CD8+ T cells numbers were positively correlated (Pearson R = 0.53, *p* < 0.0001) suggesting a common regulation of their recruitment. A key factor for macrophages and CD8+ T cells activation is CD40LG [61] and low levels of CD40LG in the tumors studied here were associated with a poor prognosis. Thus, while serum IFNγ has been recently shown to inversely correlate with tumor progression and metastasis [62], the data presented here show a broader picture of the mechanics through which IFNγ influences outcomes, by regulation of the immune cell types in the tumor microenvironment.

Among the immune checkpoint molecules analyzed, tumor necrosis factor ligand superfamily member 4 (TNFSF14) was best associated with the clinical outcome, with higher levels leading to better survival. TNFSF14 is expressed on activated T cells, activated natural killer cells, and immature dendritic cells and binds TNFSFR14 (also named Herpes Virus Entry Mediator), an important T cell costimulatory agent, also triggering IFNγ and GM-CSF release [63]. Our analysis showed that TNFSFR14 expression was also associated with better survival. Here, low levels of PD-L1 (CD274) were found to be associated with worse prognosis, while PD1 (PDCD1) was not significantly associated with overall survival. Tumor cell expression of PD-L1 is generally considered to lead to worse clinical outcomes, however, PD-L1 expression on immune cells but not on cancer cells can, at least in some cancers, predict better survival [64].

## 4. Materials and Methods

### 4.1. Data Used

All transcriptome profiling data available for breast cancer in the public database The Cancer Genome Atlas (TCGA, National Cancer Institute (NCI), Bethesda, MD, USA) were downloaded from the GDC portal (https://portal.gdc.cancer.gov/, accessed on 23 October 2022) along with the corresponding clinical information. Next, tumor transcriptomics data from patients with less than five year survival (Poor Prognosis, *n* = 101), patients with greater than five year survival, i.e., those with follow-up available for > 5 years and still alive at the last check (Good Prognosis, *n* = 200), as well as unpaired normal tissue data (*n* = 105) were extracted and used for the subsequent analysis. The workflow of the bioinformatics analysis is illustrated in Figure 1.

### 4.2. Identification of Breast Cancer Subtypes

To evaluate the HER2 and hormone receptors status, *ERBB2*, *PGR*, and *ESR1* gene data were log transformed, Gaussian-deconvoluted using the scipy package in Python3, and the intersection of the two Gaussian curves corresponding to the positive and negative receptor populations was used as threshold. The subtypes were attributed as follows: Luminal A: (ER+ and/or PR+) and HER2−, Luminal B: (ER+ and/or PR+) and HER2+, HER2 enriched: ER−, PR− and HER2+, Triple Negative: ER−, PR− and HER2−.

### 4.3. Differential Gene Expression (DEG) and Gene Ontology (GO)

The FPKM_UQ data were analyzed with unpaired t Tests, log2FoldChange values were calculated, and the p values were Benjamini-Hochberg adjusted to obtain the differential expression file. Next, clusterProfiler package in R Studio was used for gene ontology analysis, yielding a raw result containing a certain degree of redundancy. This result was further refined using REVIGO (http://revigo.irb.hr/, accessed on 26 October 2022, Ruđer Bošković Institute, Zagreb, Croatia) [65] and the top 30 enriched functions were visualized using Python 3. Target genes in the data set were identified by volcano plot visualizations of individual genes as fold change (FC) vs. adjusted *p*-value, using threshold values for both FC (0.5 and 2) and adjusted *p*-value (0.05), and labels for the most significantly modified genes and for the most over-expressed or under-expressed genes. The graphs were plotted in Python using matplotlib and adjustText libraries. We also used gene set enrichment analysis (GSEA, https://www.gsea-msigdb.org/, accessed on 2 November 2022) software from Broad Institute (Cambridge, MA, USA) [66] to identify significantly modified gene sets from the “Hallmark” (50 sets) and “C7: immunologic signature genes” (5086 sets) collections after 1% false discovery rate (FDR) correction.

### 4.4. Cell Type Enrichment Analysis

The relative abundance of different cell type in each sample group was assessed by the cell enrichment assay algorithm from CIBERSORTx (Stanfort University, Stanfort, CA, USA) [67]. The clusterplot showing the relative cell abundance in the sample groups and linking cell types that tend to correlate was graphed in Python using the Seaborn package. The relative enrichment between sample groups was assessed by ANOVA with Tukey post-test for multiple comparisons (statsmodels package in Python3).

### 4.5. Survival Analysis

Differentially expressed genes analysis revealed 2112 and 2382 genes under- and over-expressed, respectively, in PP vs. N while in GP vs. N, 2004 genes were under-expressed and 2634 overexpressed. Genes that over-expressed or under-expressed in PP but not in GP samples (*n* = 742) as well as genes coding for immune checkpoint molecules (*n* = 67) were assessed for their survival prognosis value. After grouping patients in 1/3 highest expression, center 1/3, and 1/3 lowest expression, the clinical data downloaded along with the expression files were used to assay the survival prognosis value of the selected genes, using the log rank rest included in the Lifelines Python3 package and visualizations were made using the Kaplan–Meier method from Sksurv Python3 package. The extent in which the selected 742 genes can differentiate normal tissues, PP, and GP tumors was also assayed by 2-component PCA using the sklearn package in Python.

## 5. Conclusions

By evaluation of breast cancer samples from good and poor prognostic point of view using free transcriptomic databases, we provided updates to previous reports about gene signatures in breast cancer. Starting from the whole transcriptome, we computed the differential expression, performed GO, analyzed the overall survival impact of 742 genes differentially expressed in PP and not in GP to extract the most biological significant molecules, inferred the immune cell makeup of the tumors based on the LM22 gene signature matrix, and evaluated the expression and survival impact of a further 67 immune checkpoint molecules.

Future analyses may include investigation of gene signature after adjuvant therapy, a feature that is correlated to acquired resistance to therapy. The selected gene signatures and corresponding molecules may represent starting points for future research, identification of biomarkers or molecular targets for further therapeutic approaches aiming to overcome still unsolved medical issues, such as recurrence, metastasis, or drug resistance.

## Figures and Tables

**Table 1:** Clinical data.

	Normal	All Tumors	Good Prognosis	Poor Prognosis	Good vs. Poor Prognosis *p* Value
Number	105	301	200	101	
Age					
Mean (StDev)	58.2 (14)	57.5 (13.5)	54.3 (11.7)	63.7 (14.5)	<0.0001 *** (*t* Test)
Median (Min:Max)	58 (30:90)	56 (27:90)	54 (27:85)	62 (31:90)	
Stage					
Stage I ^a^	-	43 (14.6%)	36 (18.2%)	7 (7.2%)	0.001 ** (Fisher exact test)
Stage IA	-	13 (4.4%)	11 (5.6%)	2 (2.1%)	
Stage IB	-	2 (0.7%)	2 (1%)	0 (0%)	
Stage II ^a^	-	2 (0.7%)	2 (1%)	0 (0%)	0.461 (Fisher exact test)
Stage IIA	-	83 (28.1%)	61 (30.8%)	22 (22.7%)	
Stage IIB	-	64 (21.7%)	46 (23.2%)	18 (18.6%)	
Stage III ^a^	-	2 (0.7%)	0 (0%)	2 (2.1%)	0.0047 ** (Fisher exact test)
Stage IIIA	-	51 (17.3%)	32 (16.2%)	19 (19.6%)	
Stage IIIB	-	12 (4.1%)	4 (2%)	8 (8.2%)	
Stage IIIC	-	11 (3.7%)	4 (2%)	7 (7.2%)	
Stage IV	-	12 (4.1%)	0 (0%)	12 (12.4%)	<0.0001 ** (Fisher exact test)
Subtype					
Luminal A	-	206 (68.4%)	142 (71%)	64 (63.4%)	0.191 (Fisher exact test)
Luminal B	-	18 (6%)	9 (4.5%)	9 (8.9%)	0.196 (Fisher exact test)
HER2 enriched	-	15 (5%)	11 (5.5%)	4 (4%)	0.780 (Fisher exact test)
Triple Negative	-	62 (20.6%)	38 (19%)	24 (23.8%)	0.366 (Fisher exact test)
Race					
Caucasian	98 (93.3%)	235 (78.1%)	160 (80%)	75 (74.3%)	0.302 (Fisher exact test)
African/African American	5 (4.8%)	48 (15.9%)	30 (15%)	18 (17.8%)	0.617 (Fisher exact test)
Asian	1 (1%)	10 (3.3%)	7 (3.5%)	3 (3%)	1 (Fisher exact test)

Legend: ^a^, stage without specific sub-classification in TCGA database. ** *p* < 0.01, *** *p* < 0.001.

## Data Availability

Publicly available datasets were analyzed in this study. Breast cancer RNA-seq data are available at https://www.cancer.gov/tcga (accessed on 23 October 2022). The gene sets used for gene ontology are available at https://www.gsea-msigdb.org/ (accessed on 2 November 2022), and the deconvolution matrix and the web resource for cell enrichment analysis can be found at https://cibersortx.stanford.edu/ (accessed on 30 October 2022).

## Supplementary Materials

The supporting information can be downloaded at: https://www.mdpi.com/article/10.3390/ijms24021622/s1.
