# Supplementary material for: Mining TCGA Database for Genes with Prognostic Value in Breast Cancer
Source: Int J Mol Sci. 2023 Jan 13;24(2):1622. doi: 10.3390/ijms24021622 (PMC9862022; doi:10.3390/ijms24021622)
Supplement: Supplementary file 1 [file ijms-24-01622-s001.zip › ijms-2096481_Suppl-Figures.pdf]

International Journal of
Molecular Sciences

MDPI

Supplementary Figures

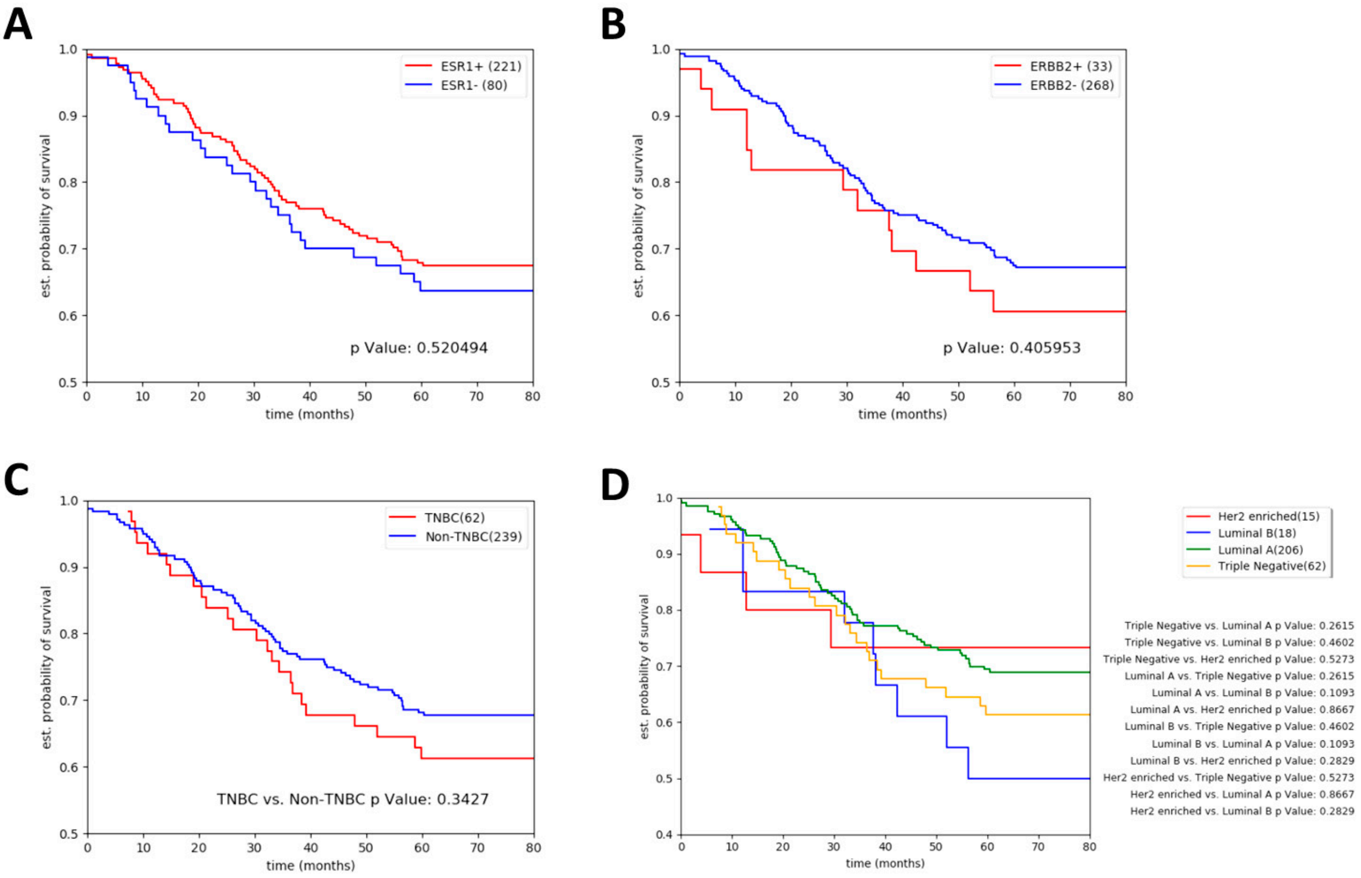

Supplementary Figure S1. Survival impact of hormone receptors status and molecular subtypes. (A) and (B) Survival curves plotted for positive vs. negative ER and HER2, respectively (C) Survival curves for TNBC patients vs non-TNBC patients. (D) Survival curves for all molecular subtypes identified. In all diagrams *p* values obtained by logrank tests are indicated.

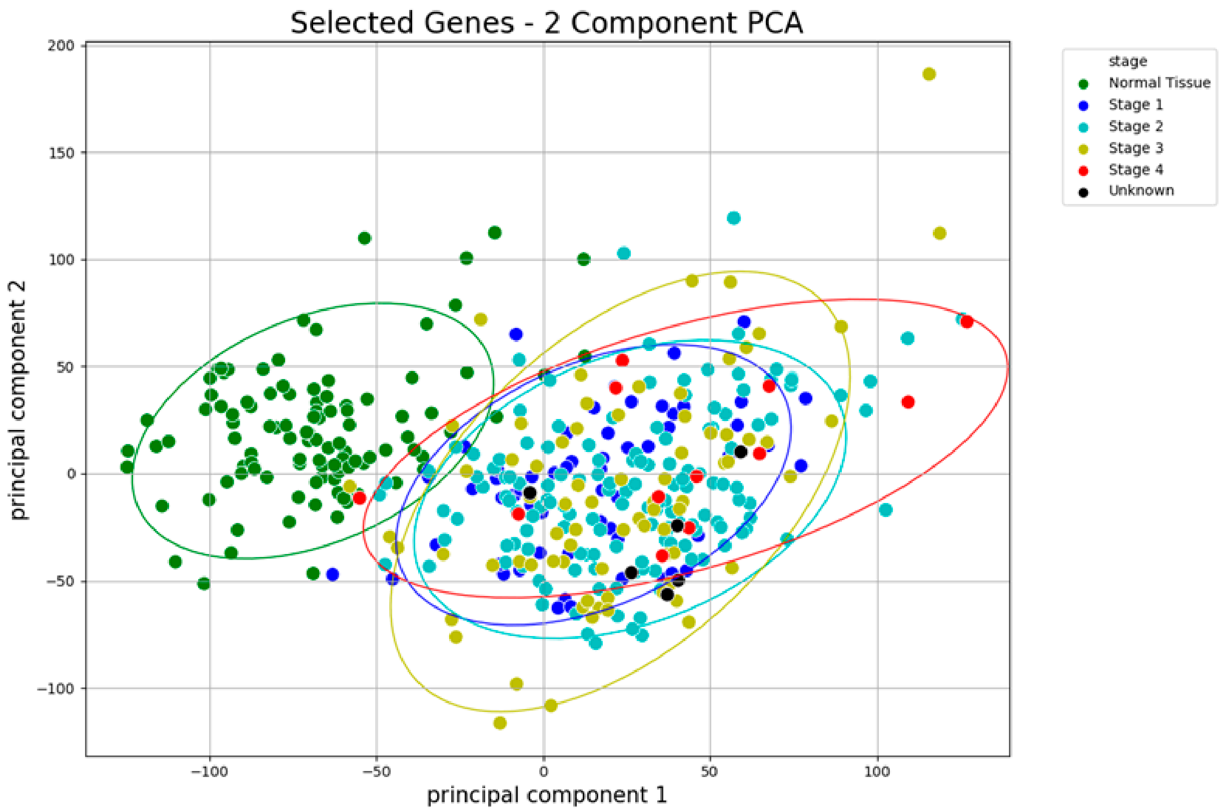

Supplementary Figure S2. Principal Component Analysis vs. Stages. Scatter plot showing the 2 principal components from a PCA performed on the 742 differentially expressed genes, with tumor stage indicated. A good separation of normal tissues from tumor tissues was observed, while tumor tissues from different disease stages overlapped.
